# Triple Negative Breast Cancer Treatment Options and Limitations: Future Outlook

**DOI:** 10.3390/pharmaceutics15071796

**Published:** 2023-06-23

**Authors:** Onyinyechi Obidiro, Gantumur Battogtokh, Emmanuel O. Akala

**Affiliations:** Center for Drug Research and Development, Department of Pharmaceutical Sciences, College of Pharmacy, Howard University, Washington, DC 20059, USA; onyinyechi.obidiro@bison.howard.edu (O.O.); gantumur.battogtokh@howard.edu (G.B.)

**Keywords:** triple negative breast cancer, chemotherapy, immunotherapy, limitations, nanotechnology

## Abstract

Triple negative breast cancer (TNBC) has a negative expression of estrogen receptors (ER), progesterone receptors (PR), and human epidermal growth factor receptors (HER2). The survival rate for TNBC is generally worse than other breast cancer subtypes. TNBC treatment has made significant advances, but certain limitations remain. Treatment for TNBC can be challenging since the disease has various molecular subtypes. A variety of treatment options are available, such as chemotherapy, immunotherapy, radiotherapy, and surgery. Chemotherapy is the most common of these options. TNBC is generally treated with systemic chemotherapy using drugs such as anthracyclines and taxanes in neoadjuvant or adjuvant settings. Developing resistance to anticancer drugs and off-target toxicity are the primary hindrances to chemotherapeutic solutions for cancer. It is imperative that researchers, clinicians, and pharmaceutical companies work together to develop effective treatment options for TNBC. Several studies have suggested nanotechnology as a potential solution to the problem of suboptimal TNBC treatment. In this review, we summarized possible treatment options for TNBC, including chemotherapy, immunotherapy, targeted therapy, combination therapy, and nanoparticle-based therapy, and some solutions for the treatment of TNBC in the future. Moreover, we gave general information about TNBC in terms of its characteristics and aggressiveness.

## 1. Introduction

Cancer continues to be a growing menace to society; in one year, a mortality rate of 8.2 million was recorded worldwide [1]. Over 13.1 million people are expected to die from cancer globally by 2030, making it the leading cause of death after cardiovascular diseases [1,2]. Cancer can affect any part of the body, occurring in all genders [2].

Breast cancer is the most commonly diagnosed cancer among women and has surpassed lung cancer, with about 2.3 million cases yearly [3,4]. This situation calls for a comprehensive understanding of the disease and the development of improved therapeutics. Breast cancer is a multifactorial disease involving environmental, hormonal, genetic, and various lifestyle and nutritional factors. Hence, breast cancer patients experience various clinical, pathological, and molecular peculiarities [5]. The molecular subtypes of breast cancer are classified by the expression profile of the estrogen receptor (ER), progesterone receptor (PR), and human epidermal growth factor receptor 2 (HER2).

**Figure 1:**
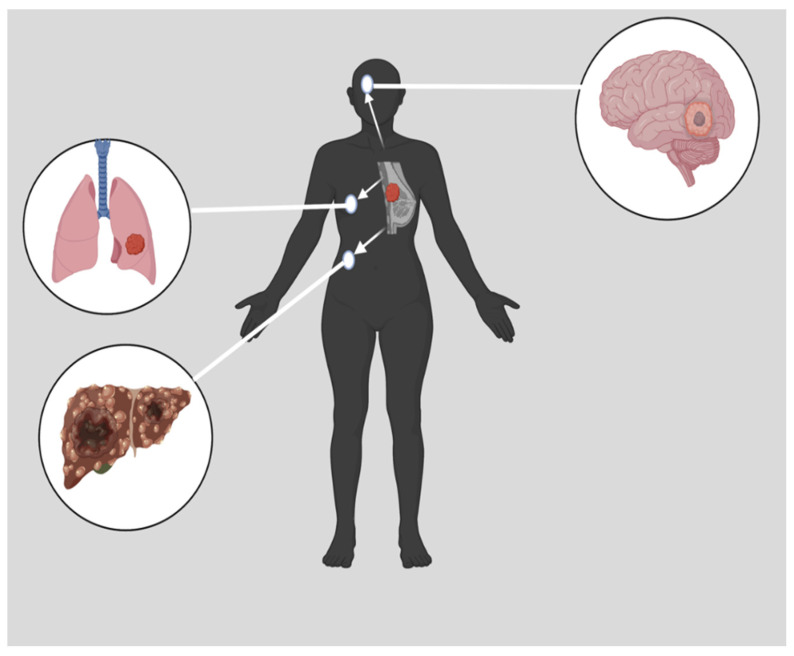
Metastasized TNBC. Many distinct characteristics are associated with TNBC. These characteristics include a high level of cell invasiveness and metastasis to other organs, such as the brain, the lungs, or the liver [10]. Created with Biorender.Com, accessed on 14 June 2023.

Triple negative breast cancer (TNBC) has low or no expression of estrogen receptors (ER), progesterone receptors (PR), and the human epidermal growth factor receptors (HER2; consequently, it does not respond to drugs that target these receptors [6,7,8,9] and there is currently no standardized TNBC treatment regimen. TNBC is characterized by a high level of cell invasiveness and visceral metastasis to organs, usually the brain, lungs, and liver (Figure 1), with an average survival time of 18 months [10,11]. TNBC is more likely to metastasize to the central nervous system and internal organs such as the liver, bones, and lungs. Patients with TNBC have a much shorter survival period once distant metastases have occurred [12]. TNBC is usually detected late in the body at the advanced stage [11,13]. It is more likely that TNBC is related to hereditary conditions than other breast cancer subtypes. In the American Cancer Society’s study of newly diagnosed breast cancer, 10% of patients had breast cancer gene 1 (BRCA1) and breast cancer gene 2 (BRCA2) mutations, whereas 35% of patients with TNBC carried a BRCA1 mutation, and 8% carried a BRCA2 mutation. There is a greater than one-third chance of developing TNBC among BRCA1 mutation carriers [14]. Typically, TNBC is diagnosed via imaging and immunohistochemistry (IHC), which are operator-dependent and time-consuming procedures [15]. To enhance the diagnostic efficiency of TNBC, rapid and advanced technologies such as immuno positron emission tomography (PET), nanobiosensor, circulating tumor nucleic acids (ctNAs), and blood-based liquid biopsy are essential [16]. Compared to other breast cancer subtypes, TNBC is the most immunogenic subtype with a limited treatment option [17].

TNBC is the most aggressive and heterogeneous of all breast cancer subtypes; hence it has been described as fatal [10,18,19]. Literature evidence indicates that it is reportedly the greatest cause of mortality in women [20,21]. TNBC accounts for 15–20% of all breast cancer. Population-based studies confirm that TNBC is prevalent in young pre-menopausal women under 40 years, African American, and Hispanic women of lower socioeconomic backgrounds [11,22,23,24]. Non-Hispanic African American women have a lower breast cancer incidence than non-Hispanic White women [25]. However, African American women have doubled triple-negative breast cancer incidence [26]. Possibly, this is due to the relatively low number of African American women receiving treatment recommended by guidelines. In addition, there is a difference in treatment outcomes between African American women and white women with TNBC. According to a population-based cohort study of 23,213 patients with TNBC, African American women were significantly more likely to die from breast cancer than white women [27]. The authors suggested that disparities in surgery and chemotherapy may contribute to this phenomenon.

There have not been many new treatment methods developed for TNBC compared to other types of breast cancer due to the aggressive nature of this type of breast cancer [28]. Chemotherapy takes advantage of the high growth rate of tumor tissues. Angiogenesis inhibitors cut the blood flow to tumor tissues, and interference in the ribonucleic acid (RNA) messenger pathway causes apoptosis in the already impaired deoxyribonucleic acid (DNA) [11,29]. There is a tendency for TNBC tissues to express more epithelial growth factor receptor (EGFR) protein than other breast cancer subtypes, which makes them more resistant to conventional treatment [7]. Several inflammatory molecules, including tumor-associated macrophages (TAM), tumor-infiltrating lymphocytes (TILs), and cytokines, reduce the immune response to TNBC during chemotherapy, increasing the possibility of complications related to TNBC. Controlling these inflammatory molecules may improve patients’ overall and disease-free survival rates with TNBC. The manipulation of microRNAs (miRNA) in TNBC can reduce the risk of early relapse and metastasis by influencing cancer cell survival, proliferation, invasion, and metastasis [28]. TNBC is associated with chemoresistance due to the poor prognosis, relapse, and distal metastasis of TNBC [30]. In this review, we discuss the current options for TNBC treatment, their limitations, and future directions for TNBC treatment.

## 2. Subtypes of TNBC

**Figure 2:**
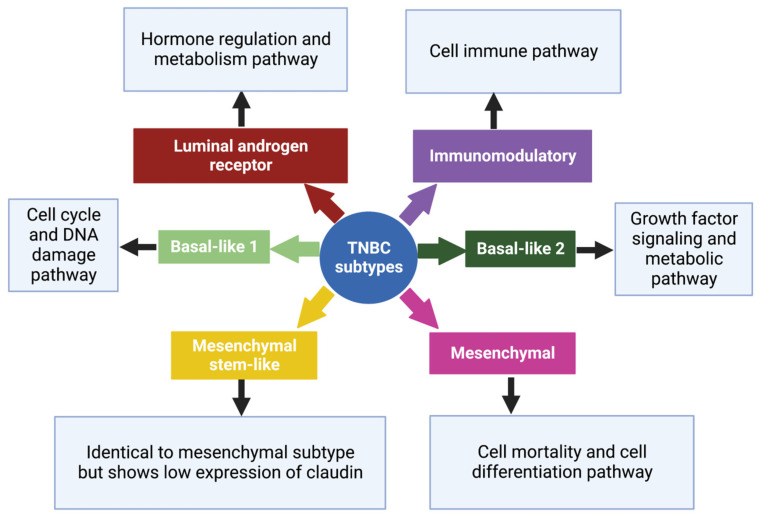
Subtypes of TNBC with their characteristic pathways. On the basis of gene expression profiles, six TNBC subtypes have been classified, each with a specific gene expression profile and ontology [28]. Created with Biorender.com, accessed on 15 June 2023.

TNBCs typically resemble basal-like breast cancer (BLBC) with overlapping gene expression characteristics [11]. Based on gene expression profiles, six TNBC subtypes, each with a different gene expression profile and ontology, were identified which are basal-like 1 (BL-1), basal-like 2 (BL-2), an immunomodulatory subtype (IM), a mesenchymal subtype (M), a mesenchymal stem-like subtype (MSL), and a luminal androgen receptor subtype (LAR) [28,31], (Figure 2). Despite this, most triple-negative breast cancers (80%) express markers of basal-associated cancer, including basal cytokeratin, vimentin, EGFR, and mutated BRCA1/2 [22]. The genetic variations among these subtypes of TNBC suggest individualized therapy rather than a generalized approach [32].

### 2.1. Basal-like 1 and 2 Subtypes

Approximately 75% of TNBCs are basal-like subtypes, so TNBC makes up the majority of these subtypes. A high level of DNA damage response is associated with the basal-like 1 subtype. According to studies, basal-like immune-suppressed subtypes of TNBC exhibit lower levels of B cell, T cell, and natural killer cells, resulting in worse prognoses. Generally, all BRCA1/2 mutations are associated with basal-like gene patterns. Anti-ER and HER2 therapies would not be effective on basal-like breast cancers since neither of these proteins is typically expressed in this type of cancer [14,33]. This subtype of TNBC is marked by high expression of cell cycle-related genes, DNA damage response (DDR) genes, and chemotherapy sensitivity [11,32]. According to studies, basal-like subtypes expressing higher levels of cell cycle genes and DNA damage response genes were sensitive to platinum drugs (cisplatin), and the BL-1 subtype has shown sensitivity to polyadenosine diphosphate (ADP)-ribose) polymerase (PARP) inhibitors [34,35,36,37].

### 2.2. Luminal Androgen Receptor Subtype

The luminal androgen receptors regulate the synthesis of steroids and porphyrin, as well as the metabolism of androgens and estrogens. In most TNBCs, androgen receptor (AR) expression is associated with a high survival rate. AR is, therefore, a prognostic indicator. This subtype has a 10-fold higher androgen receptor expression than other subtypes. The findings of recent studies suggest that AR inhibitors could be beneficial to patients with TNBC who are AR-positive [14,38].

### 2.3. Mesenchymal and Mesenchymal Stem-like Subtypes

In addition to the mesenchymal subtypes featuring pathways involved in cell motility and differentiation, the mesenchymal stem-like subtype has components interfering with the EGFR, calcium signaling, and G-protein receptors. It is characterized by elevated expression of signal transducer and activator of transcription (STAT) genes responsible for developing T-cells, B-cells, and natural killer cells. Various changes are observed in the M subtype, influencing extracellular matrix (ECM) receptor interaction. Epithelial–mesenchymal transformation (EMT) and cancer stem cells are characteristic of the M subtype [39]. Phosphatidylinositol 3-kinase/mammalian target of rapamycin (PI3K/mTOR) inhibition and non-receptor tyrosine kinase (Src) inhibition were effective in suppressing the epithelial–mesenchymal transition (EMT) and growth factor pathways in mesenchymal and MSL cell lines [34].

### 2.4. Immunomodulatory Subtype

This subtype shows similarity to the basal-like subtype. The prognosis in this subtype is favorable, but the histological grade is high. These subtypes are immune-evading due to a saturation of immune cell signaling. These tumors likely achieve immune escape by recruiting immune suppressive cells or activating immune checkpoint molecules, which provides a potential reason to use an immune checkpoint blockade therapeutically [32].

## 3. Prognostic Biomarkers

Prognostic markers of TNBC must be identified to develop effective and precise agents targeting the disease. Some of the factors that can help predict the outcome of TNBC include node status, cathepsin D, the Ki67 index, the promoter methylation value of BRCA1, and p53. These genes regulate cell proliferation (c-erbB-2 and c-erbB-3), cell death (p53), cell differentiation (pS2, ERα, and PgR), and cell invasion (cathepsin D) [7]. Moreover, there is a higher level of vascular endothelial growth factor (VEGF), TILs, and TAM in TNBC [40]. Usually, these predictive tools can accurately predict disease-free survival and overall survival rates in patients with TNBC, and if the prognosis is poor, other treatment options might be considered [28].

In the current era of cancer research, systemic immunotherapy is a treatment option for TNBC since this type of cancer has been proven to be immunogenic, as evidenced by stromal tumor-infiltrating lymphocytes (STILs), a prognostic and predictive marker. Patients with TNBC also express a high level of tumor-infiltrating lymphocytes [41], which have been demonstrated to be useful prognostic indicators. Compared to other subtypes, TNBC has consistently elevated TILs, which are associated with better survival [30]. The pathological complete response (pCR) rate in lymphocyte-predominant breast cancers was 40%, compared to 7% in non-lymphocytic cancers [40,41]. The results of studies with checkpoint inhibitors [21] have shown the importance of immune marker assessment in TNBC. TILs are predictive and prognostic, especially in TNBC [42]. Breast cancers associated with BRCA1 exhibit high mitotic index, p53 mutations, and triple-negative characteristics [43,44]. Alli and co-workers used a BRCA1 murine mammary epithelial cells (MMECs) model to examine the effect of BRCA1 gene loss on cellular sensitivity to various chemotherapy drugs. Despite not being included in first-line regimens for breast cancer, cisplatin and gemcitabine show therapeutic effectiveness in BRCA1-deficient MMECs. Even so, standard breast cancer therapy agents such as doxorubicin, 5-fluorouracil, paclitaxel (PTX), and docetaxel were also effective [45].

There is a relatively high tumor mutational burden (TMB) in TNBC. Each TNBC has approximately 60 somatic mutations per megabase (Mb) of coding regions. There is an uneven mutation burden in TNBC, with some tumors having a high mutation burden (more than 4.68 somatic mutations per Mb) and multiple copy-number aberrations involving genes that alter multiple pathways. In malignancies, TMB has been identified as a possible biomarker for TNBC. TMB enhances the immune response by increasing neoantigen expression and presentation. There has not been sufficient clinical evidence for TMB to enter routine practice. The United States Food and Drug Administration (FDA) recently approved pembrolizumab for tumors displaying a high TMB [40]. AR expression correlates with a good survival rate in most TNBC; this should be considered when evaluating ER, PR, and HER2 status, especially in African American women [39]. Table 1 highlights common predictive biomarkers in TNBC.

## 4. Treatment Strategies for TNBC

TNBC is characterized by the lack of ER, PR, and HER2. TNBC is a heterogeneous and aggressive cancer for which no effective biologically targeted treatment has been approved. There are several challenges to TNBC treatment, including poor prognosis, tumor heterogeneity, chemotherapeutic side effects, and the possibility of metastasis [51]. TNBC is extremely difficult to treat due to the absence of ERs, PRs, and HER2; however, the biological and pathological characteristics of TNBC provide insight into several potential molecular targets for current and future therapeutics [28]. The triple negative paradox refers to TNBC being intrinsically chemo-sensitive and prone to rapid relapse and resistance [40]. Chemotherapy with anthracycline/taxanes has been the standard of treatment in systemic neoadjuvant and adjuvant therapy in all newly established cases [18]. The goal of neoadjuvant therapy is to shrink the tumor before the main treatment, whereas adjuvant therapy is given after the main treatment.

**Figure 3:**
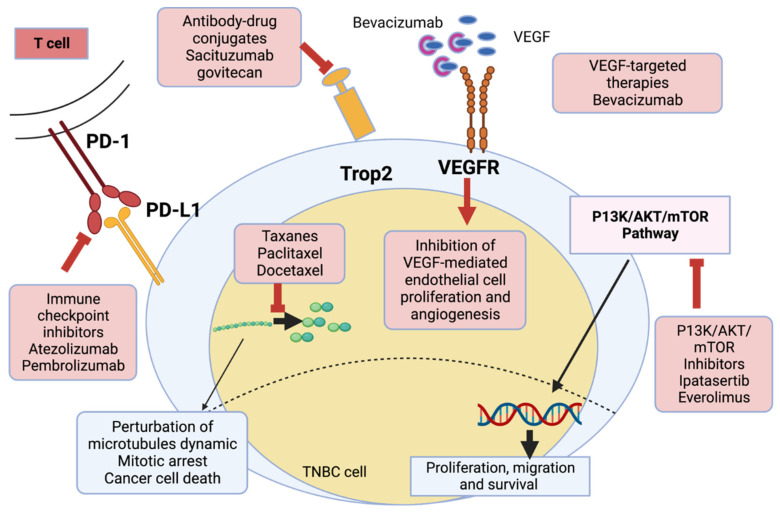
Various triple negative breast cancer (TNBC) treatments available in the clinic. As far as therapeutic strategies are concerned, several approaches have been proposed for both patients with early and advanced triple negative breast cancer (TNBC). These include immunotherapy, deoxyribonucleic acid (DNA)-interfering agents, and targeted therapies [53]. Created with Biorender.com, accessed on 14 June 2023.

A doctor’s decision-making in selecting and prescribing the appropriate treatment regimen is usually based on clinical and pathological parameters such as the patient’s age, the stage (TNM; tumor size, lymph node status, metastasis), the tumor grade, the histology of the tumor, and the molecular subtype of the breast tumor. It was evident in the study by Rouzier and his group that the response to preoperative chemotherapy depends on the molecular subtypes of breast cancer [52]. In another study, TNBC survival was predicted by Polley et al. based on a cohort [18]. Here, it was demonstrated that a clinical decision tool for early-stage TNBC has been developed and authenticated, which will increase our understanding of predictive factors. In addition to helping with individualized disease prognostication and treatment planning, their study sets the stage for future studies to incorporate additional biomarkers into outcome estimates. TNBC patients are treated with various therapeutic interventions, depending on their type and stage of cancer [53]. Some of these interventions have been highlighted in Figure 3 and include chemotherapy, immunotherapy, radiotherapy, stem cell therapy, laser treatment, hyperthermia, surgery, and photodynamic therapy [7,54,55]. Out of these, chemotherapy is the most common. Figure 3 describes clinically available treatment options of TNBC.

Early-stage and low-risk TNBC require considering the balance between risks and benefits when designing the optimal treatment sequencing and therapy combinations. In either case, chemo-toxicity and poor outcomes may result from over- or under-treatment [56]. TNBC is known for metastasis, mutational capacity, increased TILs, and programmed cell death. These make TNBC susceptible to immunotherapy and chemotherapy [8,57,58,59]. The heterogeneousness of TNBC suggests various phenotypes which serve as more targets for TNBC: basal 1, basal 2, immunomodulatory, luminal androgen receptor, mesenchymal, stem cell, and unstable [11,57,60]. Based on gene expression profiling, BRCA1/2 gene mutations are implicated in TNBC [4]; thus, PARP inhibitors, such as olaparib and talazoparib, are used as therapy for breast cancer caused by these mutations [61]. Overall, nanotechnology-based approaches to treating breast cancer in general with combination chemotherapy will offer numerous advantages in modern medicine [62].

### 4.1. Surgery

TNBC is treated by surgery. A lumpectomy (removal of the tumor and a small amount of surrounding tissue) or a mastectomy (removal of the whole breast) can be performed depending on the stage of the cancer [63]. Approximately 20,000 women were analyzed in a meta-analysis study to resolve the controversy over patient outcomes following breast conservation therapy or mastectomy [64,65]. Study results indicate that women with TNBC who undergo breast-conserving therapy do not have a worse prognosis than those who undergo mastectomy. When clinically feasible, breast-conserving surgery followed by radiotherapy may be routinely offered to women with TNBC. There is also the possibility of removing lymph nodes. It is possible to undergo surgery before hormonal therapy with AR or chemotherapy. Surgery is common for locoregional or sentinel lymph node metastatic breast cancer (MBC). Depending on the clinical condition and patient characteristics, surgery can be used alone or with chemotherapy to improve MBC treatment efficiency. Moreover, surgery can help patients survive and reduce their mortality by avoiding potentially disabling complications (medullary compression, pathologic fractures), resecting metastases (lungs, ovary, and liver), treating the symptoms (chest wall infiltration, local recurrence, and bone pain), and excluding other tumors and non-tumor diseases [66]. Surgery, however, can increase peripheral oxidative damage to macromolecules in the early postoperative period, so antioxidant supplementation is recommended [7].

### 4.2. Radiation

There is a greater risk of recurrence in TNBC patients than in those with other subtypes. Moreover, it is known that TNBC has distinct features, including occurrence in young people, aggressive morphological characteristics, and worse outcomes that do not always correlate with traditional prognostic indicators. Additionally, TNBC patients seem to develop distant and regional recurrences at a higher rate than their non-TNBC counterparts [11,13]. Often, radiation therapy is used after mastectomy to destroy the remaining cancer cells in the lymph nodes and breast [66]. Moreover, it can be used to treat cancer that has spread. Radiation, however, can lead to relapses in 7–12.6% of patients within 5 years, and resistance can also develop; therefore, radiotherapy and immunotherapy are commonly used together [67,68]. In addition, trials are being conducted on TNBC to manipulate the immune system with radioimmunotherapy since it lacks expression of other molecular targets to improve outcomes [67]. A phase 1 study that combines pembrolizumab with intraoperative radiation therapy (IORT) is currently being conducted under an open-label, single-arm, single-institution conditions in newly diagnosed triple-negative, node-negative breast cancer patients (NCT02977468). Before enrollment in this study, subjects may not have received neoadjuvant chemotherapy, definitive surgery, or radiation therapy. By treating TNBC patients with MK-3475 (pembrolizumab), the project intends to determine if immune modulation will alter the expression of immune-tolerant markers. In this study, researchers will examine how pembrolizumab affects breast stromal responses to high-dose radiation delivered by IORT [69].

### 4.3. Chemotherapy

A combination of chemotherapy and surgery is typically used for patients with TNBC, as it is a highly effective method of killing cancer cells throughout the body. Chemotherapy regimens vary depending on the patient and cancer stage. Systemic chemotherapy is the standard for TNBC treatment and uses drugs such as anthracyclines (e.g., doxorubicin) and taxanes (e.g., paclitaxel) in the neoadjuvant (NACT) or the adjuvant setting [43,70]. The first NACT was performed in the 1980s on locally advanced breast cancer patients to make inoperable tumors operable [71]. Although the first-line treatment for TNBC appears to be chemotherapy, studies suggest it should be applied based on tumor size. Gupta et al. observed that TNBCs with tumor sizes between 0.5 cm and 1.0 cm that did not involve lymph nodes have a good prognosis [56]. Another group of researchers opined that adding chemotherapy to TNBC tumors that were less than 1 cm in diameter was not significantly beneficial [72]. With chemotherapy alone, small tumors can achieve complete pathologic remission. Patients with tumors stage IIA and higher tended to have a higher pCR rate with durvalumab than with placebo. This was demonstrated in a randomized, double-blind, placebo-controlled phase II trial investigating the pCR rate of NACT, including nab-paclitaxel followed by dose-dense epirubicin/cyclophosphamide with durvalumab versus placebo in primary non-metastatic TNBC patients [73].

#### 4.3.1. Anthracyclines

The standard chemotherapy regimen for TNBC usually consists of anthracyclines and taxanes [56]. Through intercalation, anthracyclines destabilize DNA to cause DNA repair cascade degradation, which can benefit TNBC [35]. Research suggests that anthracyclines kill cancer tissues directly and activate the immune system by activating CD8+ T cells [74]. It has been shown that anthracyclines such as doxorubicin and epirubicin enhance response rates and survival by several months. Whether used as a separate agent or as a neoadjuvant, these agents have proven beneficial and improved sensitivity. Patients with wild-type BRCA1/2 who have not received these agents in neoadjuvant or adjuvant settings are most often treated with anthracyclines or taxane-based regimens. Although it is evidence-based that patients may respond to re-challenge with these agents, many physicians are opposed to re-challenge in the case of anthracyclines due to cumulative cardiac toxicity [40].

Additionally, anthracycline-based therapy indeed has a higher response rate. Still, it is also associated with higher recurrence rates and a low overall survival rate, which restricts its use. The drug can also cause acute toxicity, such as irreversible cardiotoxicity, myelotoxicity, alopecia, nausea, and vomiting [75].

#### 4.3.2. Taxanes

The unique mechanism of action of taxanes and their wide applications in cancer therapy make them a significant class of anticancer agents. Key molecular mechanisms of taxanes include disruption of the mitotic spindle, mitotic slippage, and inhibition of angiogenesis [76]. There is evidence that either taxanes alone or combined with anthracyclines can benefit TNBC compared to other subtypes of breast cancer, with pCR rates at least 40% [75,77]. Three taxanes have been approved for clinical use: paclitaxel, docetaxel, and cabazitaxel. As a chemotherapeutic medicine, docetaxel (Taxotere^®^), approved in the 1980s, is one of the most effective drugs in the treatment of cancer, but it also may lead to antibiotic resistance owing to its adverse side effects on the normal microbial flora in the body [53].

Among the chemotherapeutic agents, paclitaxel is highly regarded because of its specific, reversible, and saturable binding properties to microtubules and macromolecules. PTX causes cell death in tumor tissues by inhibiting mitosis. It has a strong chemotherapeutic capacity for many cancers, such as breast, prostate, and ovarian [78]. The anticancer activity of paclitaxel is demonstrated in treating ovarian, breast, lung, Kaposi’s sarcoma, bladder, prostate, esophageal, head and neck, cervical, and endometrial cancers [79]. However, the apoptosis of PTX can experience limitations, including the cell’s use-dependent resistance and toxicities to healthy cells; therefore, there is a need for combination therapy to treat TNBC effectively [80]. Albumin-bound paclitaxel (nab-paclitaxel) was developed to improve the drug’s safety profile.

Futamura et al. performed a meta-analysis to determine the safety of nab-paclitaxel. The team evaluated the average effectiveness of nab-paclitaxel-containing regimens as neoadjuvant chemotherapy in patients with operable breast cancer using individual patient data (IPD). pCRs rates of each breast cancer subtype were the primary endpoints. According to the results, nab-paclitaxel is safe and effective in neoadjuvant settings, especially for aggressive cancer [81]. Another study by Yardley and coworkers demonstrated that combined chemotherapy regimens containing platinum or taxane might treat metastatic TNBC (mTNBC). In this study, the investigators evaluated the efficacy and safety of first line nab-paclitaxel plus carboplatin (nab-P/C), nab-paclitaxel plus gemcitabine (nab-P/G), and gemcitabine plus carboplatin (G/C) for the treatment of metastatic TNBC. The primary outcome was progression-free survival (PFS); the secondary outcomes were overall response rate (ORR) and overall survival (OS). Compared with nab-P/G or G/C, nab-P/C had a significantly longer PFS and improved risk/benefit profile in mTNBC. With nab-P/C, PFS was significantly longer than with nab-P/G (hazard ratio (HR), 0.59 (95% CI, 0.38–0.92); *p* = 0.02) or G/C (HR, 0.58 (95% CI, 0.37–0.90); *p* = 0.02). In nab-P/C, OS was numerically longer than in nab-P/G (median, 16.8 versus 12.1; HR, 0.73 (95% CI, 0.47–1.13); *p* = 0.16) or G/C (median, 16.8 vs. 12.6; HR, 0.80 (95% CI, 0.52–1.22); *p* = 0.29). There were 73%, 39%, and 44% ORR, respectively [82].

#### 4.3.3. Platinum

The effects of platinum agents on DNA strand breaks and apoptosis are well known; their unique mechanism of action makes them particularly effective against cancer tissues with defective DNA repair mechanisms, including those that carry deleterious BRCA variants [83,84]. DNA repair defects characterize TNBC. It is known that platinum and anthracyclines (as well as cyclophosphamide) induce DNA damage directly, which is why these agents have been highly considered in metastatic TNBC and especially in those with germline BRCA mutations (gBRCAm). Many questions have been raised about whether BRCA1/2 germline variants are associated with platinum therapy sensitivity. gBRCAm and BRCAness statuses are associated with increased sensitivity to chemotherapy and better outcomes [85]. “BRCAness” refers to tumors that lack germline mutations in BRCA1/2 but have similar descriptions as BRCA-mutated tumors [14]. Patients with BRCA1/2 mutant TNBC and other defects in homologous recombination have shown significant benefits from platinum-based regimens [40]. A phase III study equating carboplatin with docetaxel in TNBC patients with mutated BRCA1/2 demonstrated the benefit of platinum agents for treatment in TNBC patients with germline BRCA1/2 mutations. This study’s primary outcome was an ORR. According to the trial results, carboplatin was not more effective than docetaxel in the unselected population of 376 patients (ORR: 31.4 vs. 34.0; *p* = 0.66). Contrary to this, gBRCAm- breast cancer patients treated with carboplatin had twice the ORR compared with docetaxel (68% vs. 33%) [85].

Studies have demonstrated that adding platinum to an NAC regimen increases pCR rates [86,87,88,89,90]. Given the accruing evidence demonstrating improved long-term outcomes among patients who achieve a pCR, recent systemic therapy efforts for TNBC have focused on adding agents likely to improve pCR rates. The most notable examples are platinum and immunotherapy [86,90]. Dose-dense schedules and platinum should be considered in the NACT setting. For patients without a pCR, capecitabine is an option to improve the outcome. Platinum agents are effective in increasing pCR when added to anthracycline/taxane-based chemotherapy but at a potential cost of increased toxicity. Researchers found that paclitaxel-plus-carboplatin may be an alternative adjuvant chemotherapy approach for patients with operable TNBC compared with anthracycline-docetaxel regimens [83]. The use of carboplatin, with or without PARP inhibitors, as part of neoadjuvant chemotherapy in patients with TNBC has been controversial, even though randomized trials suggest it increases the likelihood of achieving a complete pathological response. Following the supplement of carboplatin to neoadjuvant chemotherapy for patients with high risk, TNBC has a favorable risk-to-benefit profile based on results from a randomized, double-blind, placebo-controlled trial [91].

### 4.4. Targeted Therapy

#### 4.4.1. PARP Inhibitors

PARP inhibitor was approved recently for treating metastatic breast cancer in people with BRCA mutations [92]. It was investigated whether PARP inhibitors could treat BRCA-deficient cancers [45,93]. The BRCA1/2 genes are essential in homologous recombination repair (HRR) to repair DNA damage. These genes play a major role in repairing DNA lesions that cause double-strand breaks (DSB) and stall replication [94]. Despite many conceivably distinct forms of HRR in eukaryotes, the proper form requires BRCA2 to bind directly to RAD51 and localize it to damaged DNA; without functional BRCA2, HRR is impaired. Although BRCA1-mutant cells have impaired RAD51 localization, the role BRCA1 plays in DNA damage response appears broader since BRCA1 controls HRR signal transduction and determines whether double-strand breaks are removed before forming RAD51 nucleoprotein. Women who carry heterozygous germline mutations in BRCA1 or BRCA2, which impair their function, have increased risks of breast, ovarian, and other cancers. Based on this BRCA1/2 biology, a therapy for TNBC that targets HRR defects, notably PARP inhibitors, has been developed [95,96]. Some PARP inhibitors are currently FDA-approved for use in the clinic, even though BRCA mutations account for only 20% of the cancer population. These include olaparib, veliparib, niraparib, rucaparib, and talazoparib. A clinical trial for veliparib and niraparib in TNBC is currently underway, but FDA approval has been granted for olaparib and talazoparib in BRCA-mutant TNBC [37,75,97,98,99]. Cells with gBRCAm are sensitive to PARP inhibition due to the synthetic lethality mechanism, resulting in an incapacity for DNA repair. As gBRCAm is related to a higher pCR rate and a prognostic impact in patients with TNBC, assessment of the gBRCAm status should be contemplated in the early setting. Pluripotent cancer stem cells are thought to play a prominent role in primary malignant solid tumors. In addition to forming drug-resistant proteins, cancer stem cells are also associated with metastasis. In light of this, PARP inhibitors are one of the most promising therapies currently being investigated for BRCA1/2 mutations in TNBC [92]. However, reversing BRCA mutations and other mechanisms reduce PARP inhibitor’s efficacy, and only a fraction of patients with TNBC have BRCA mutations, limiting their effectiveness. Therefore, advanced therapies are needed to sensitize TNBC regardless of BRCA mutation status [100].

#### 4.4.2. Androgen Receptor Antagonists

TNBC may lack the conventional hormonal receptors associated with breast cancer, but there are other hormone receptors in TNBC, such as glucocorticoid and androgen receptors (AR). There is a 12% to 36% expression of AR, a nuclear hormone transcription factor activated by ligands [101]. ARs are important components of TNBC cell proliferation, invasion, migration, and apoptosis, all of which can lead to disease complications. TNBC may benefit from treatment using AR inhibitors that target AR [28]. In patients with TNBC, AR, a type of steroid hormone receptor, has recently been found to be a prognostic and treatment-predictive marker. A significant portion of patients with invasive breast cancer and TNBC have AR. AR expression levels in TNBC vary greatly. Agents targeting AR may be the best treatment markers for TNBC in patients with AR-dependent TNBC compared to those with AR-independent TNBC [102]. According to one study, anti-androgen drugs may be a reliable treatment marker for patients with AR-positive and triple negative TNBC. Researchers examined abiraterone acetate (AA) in combination with prednisone in women with AR+ and ER-, PR-, HER-2- metastatic or inoperable locally advanced breast cancer in a multicenter, two-stage study. After six months, results indicated that AA plus prednisone was effective for patients with AR-dependent TNBC, suggesting that targeting AR may be a potential treatment strategy [28]. It was assessed in a phase II study that patients with locally advanced or metastatic AR-positive TNBC received 160 mg of enzalutamide once daily until disease progression. The primary outcome was the clinical benefit rate after 16 weeks of treatment. A secondary outcome included progression-free survival at 24 weeks and safety. Enzalutamide was well tolerated and demonstrated clinical activity in patients with AR-positive TNBC. Enzalutamide’s safety profile was consistent with its known side effects. The findings of this study support further development of enzalutamide for advanced TNBC [103]. Currently, enzalutamide is in a clinical trial as a targeted AR inhibitor that inhibits translocation to the nucleus by competitively binding to the AR [75].

#### 4.4.3. EGFR Inhibitors

EGFR is a promising biological target for cancer therapy. It is a cell surface transmembrane tyrosine kinase receptor that regulates cell proliferation, differentiation, invasion, angiogenesis, and apoptosis through various signaling pathways and is a poor prognostic factor for cancer [48]. A high level of EGFR expression is associated with large tumors, poor differentiation, and poor clinical outcomes in breast cancer [104]. Several studies have shown that basal-like TNBCs overexpress the EGFR gene (27–57%). Several factors can lead to excessive signaling, including receptor overexpression, autocrine signals, or mutations. Mammalian cells are controlled by EGFR signaling for growth, survival, proliferation, and differentiation. It has been found that TNBC displays a higher expression of EGFR protein and mRNA than normal tissue [105]. Normal cells usually express 4 × 10^4^–1 × 10^5^ of EGFR per cell, but cancer cells can express as many as 2 × 10^6^. EGFR receptors are, therefore, suitable for targeted therapy [21]. Therefore, suppressing the EGFR protein potentially enhances the efficacy of TNBC treatment. Small interfering RNAs (siRNA) targeted to EGFR messenger RNA (mRNA) can be used to achieve this goal. The naked siRNA, however, is unstable in the bloodstream or within cancer tissues.

Additionally, it has very poor penetration abilities inside cancer tissues. Nanotechnology has proven effective in delivering siRNA and conventional anticancer therapies inside TNBC [106,107]. To provide efficient intracellular drug delivery, immunoliposomes targeting EGFR and its variants have been considered [108]. EGFR inhibition as a therapeutic option in TNBC treatment has been investigated. Even though TNBC cells are less responsive to EGFR inhibition than HER-2-positive cell lines, gefitinib enhances their response to chemotherapy. Cetuximab is an EGFR inhibitor approved by the FDA. Combining gefitinib, carboplatin, and docetaxel may be beneficial in treating TNBC. In clinical trials, the small molecule inhibitors gefitinib and erlotinib, and monoclonal antibody cetuximab are currently used as EGFR inhibitors of solid tumors [109].

#### 4.4.4. VEGF Inhibitors

VEGF receptor (VEGFR) is a part of the receptor tyrosine kinases (RTK) family of proteins. A receptor tyrosine kinase is an enzyme-linked transmembrane receptor consisting of a transmembrane helix, an extracellular binding region, and a protein tyrosine kinase domain [110]. VEGF stimulates the proliferation and growth of tumor cells and the formation of new vessels in growing tumors. It plays a significant role in breast cancer development. There is an association between higher VEGF levels in TNBC and poor outcomes regardless of tumor size, nodal status, or histological grade [56]. Angiogenesis is considered key to tumor cell proliferation, so bevacizumab, a monoclonal antibody that binds the VEGF receptor, has been investigated and is FDA-approved as a treatment for TNBC. An increase in pCR was observed with bevacizumab added as a neoadjuvant treatment [47].

### 4.5. Immunotherapy

There has been an increase in immunotherapy research in cancer biology and oncology. While immunotherapy does not have a robust response in general to breast cancer patients, it has been shown that a subset of TNBCs has high TMB and high TILs, like those observed in melanoma or lung cancer, which may be treated with immune checkpoint inhibitors. Consequently, developing immunotherapies that target TNBC has become possible due to its immunogenic nature [111]. Recently, the FDA approved atezolizumab combined with nab-paclitaxel for treating metastatic and unresectable programmed cell death ligand 1 (PD-L1)-positive TNBC [112]. The body uses the immune system, especially the T cells, to fix cellular abnormalities. Cancer cells, however, use various strategies to avoid any immune destruction. The PD-L1 protein is overexpressed on tumor cells and binds to T cell receptors programmed cell death proteins 1 (PD-1) and CD80 (B71). T cells then receive an inhibitory signal; subsequently, T cells become exhausted and ineffective.

#### 4.5.1. Immune Checkpoint Inhibitors (ICIs)

Systemic immunotherapy directly utilizes the patient’s immune system to eradicate and target neoplastic tissues [73,74]. According to the Cancer Genome Atlas, TNBC has higher PD-L1 mRNA expression. Hence, PD-1 and PD-L1 inhibitors are currently being researched in early-stage and MBC. TNBC also had higher levels of CD8+ T cell infiltration. To boost T cell activity, many approaches have been devised to achieve a more intensive cytotoxic effect and, consequently, a faster death of tumor tissues [74]. By inhibiting the binding of PD-L1 to PD-1, T cell exhaustion is reversed, and antitumor responses are strengthened. Studies are being conducted on several monoclonal antibodies in the setting of TNBC [113,114]. The antibodies targeted at PD-L1 include atezolizumab, durvalumab, and avelumab, while those targeting PD-1 consist of nivolumab and pembrolizumab. Monoclonal antibodies such as pembrolizumab and nivolumab bind to PD-1, preventing the interaction between PD-1 and its ligand, PD-L1. As a result, the immune response is not evaded but rather upregulated to help kill abnormal tumor cells. The FDA has approved pembrolizumab and nivolumab for multiple solid tumors, and several studies are examining their effectiveness in treating TNBC [114,115,116]. Wu et al. evaluated ICIs as a potential therapy for TNBC. The study included patients with metastatic or inoperable CD8+ TNBC without prior therapy. Using immunohistochemistry, CD8+ disease was defined as the presence of CD8 in 10% of cells. Angiogenesis inhibitor famitinib, PD1 monoclonal antibody camrelizumab, and chemotherapy were assessed in this study for combination treatment of advanced immunomodulatory TNBC. 81.3% of patients achieved an objective response (95% CI, 70.2–92.3), and a median progression-free survival of 13.6 months (95% CI, 8.4–18.8) was reported within 48 patients. There were no treatment-related deaths. According to these results, patients with CD8-positive and PDL1-positive tumors are more likely to benefit from this regimen [114].

Another regulatory pathway of T cells is cytotoxic T lymphocyte-associated protein-4 (CTLA-4). The CTLA-4 gene is upregulated during T cell activation and competes with the CD80/CD86 gene to inhibit intracellular T cell activation signaling, inhibiting the immune response. The inhibition of CTLA-4 enhances the activation of T cells. The monoclonal antibody tremelimumab targets CTLA-4 and is under investigation for TNBC treatment [74]. Tremelimumab inhibits cytotoxic T lymphocyte-associated protein-4 (CTLA-4). T cells cannot activate if CTLA-4 is bound to CD80/CD86 on their surface. By blocking this interaction, more T cells are activated, leading to greater cancer cell death. The FDA has already approved CTLA-4 inhibitors for other solid tumors but not yet for TNBC [11,106]. As stromal tumor-infiltrating lymphocytes (STILs) (an indicator of prognosis and predictive accuracy) are evidence that TNBC is immunogenic, systemic immunotherapy is an option for treating it. The results of studies with checkpoint inhibitors [20] have shown the importance of immune marker assessment in TNBC. There is ongoing research into immune vaccines, checkpoint inhibitors, and other potential treatments for TNBC. The reader is directed to Katz et al. [74] for more details on monoclonal antibodies under investigation for TNBC treatment.

#### 4.5.2. Antibody-Drug Conjugates

Antibody-drug conjugates (ADC) provide an ideal delivery method for cytotoxic payloads to treat different cancers. A monoclonal antibody can deliver a drug to a specific target [117]. Depending on their purpose, they may be used alone, combined, or conjugated with bioactive agents or other delivery systems, including micelles, polymeric nanoparticles, liposomes, etc., to target specific sites for delivery [118]. Cancer therapies are still hampered by resistance to cancer cells. Cancer cells become resistant to therapeutic pressures through various mechanisms. Secondary resistance to the drug may have developed after treatment (secondary or acquired resistance) or may have developed from the beginning of the treatment (primary or de novo resistance). It is generally accepted that resistance mechanisms to ADC are caused by each component of the ADC, namely monoclonal antibodies, cytotoxic drugs, or survival signals. As part of an ADC system, antigen-related resistance involves changes in the antigen levels recognized by the monoclonal antibody component. Multiple cycles of ADC treatment have been demonstrated to decrease target antigen levels in cancer cell lines exposed to various ADCs. Liu et al. found that doxorubicin loaded in cetuximab–DNA conjugates were more effective in cytotoxicity of EGFR-overexpressed cell lines than doxorubicin in free form, which suggests that the conjugates were more readily and selectively taken up by cells, where doxorubicin was released from endosomes rapidly into the cytoplasm [119]. Sacituzumab govitecan (SG) was approved by the FDA in 2016 to manage metastatic triple-negative breast cancer patients who have had at least two previous therapies [120]. SG combines a humanized anti-Trop-2 antibody and SN-38, a metabolite of irinotecan. When SG is administered, the anti-Trop-2 monoclonal antibody binds to Trop-2 expressed on tumor cells, enabling internalization and targeted delivery of SN-38. SN-38 can be released into the tumor microenvironment, eliciting antineoplastic effects without internalization and enzymatic cleavage from anti-Trop-2 antibodies [51,88,121].

#### 4.5.3. P13/AKT/mTOR Inhibitors

Autophagy inducers, nuclear factor kappa B (NF-_k_B), and mTOR have also contributed to disease progression. Inhibitors of PI3K, AKT (protein kinase B), and mTOR can be considered for treating TNBC whose molecular status has been altered [28]. The metabolic, proliferation, survival, growth, and angiogenesis processes are controlled by PI3K and AKT, which operate in response to extracellular signals. In a study, AKT inhibitors, such as ipatasertib, were utilized with PTX to improve progression-free survival [75]. Despite high levels of activated PI3K/AKT pathways in TNBC, complexities are associated with this pathway. PI3K/AKT/mTOR inhibitors have not yet been approved for TNBC. Nonetheless, some PI3K/AKT/mTOR inhibitors are being studied for treating TNBC. The FDA approved everolimus, an mTOR inhibitor, as a treatment for postmenopausal women with advanced hormone receptor-positive HER2-positive breast cancer. In an ongoing study, everolimus is under study for the treatment of advanced TNBC (NCT05563220) [110,122]. Other P13/AKT/mTOR inhibitors under study for TNBC are PI3K inhibitors (buparlisib and taselisib), AKT inhibitors (capivasertib, ipatasertib), and mTOR inhibitors (everolimus and temsirolimus) [123,124,125].

### 4.6. Combination Therapies

Single agents versus combination therapies have been the subject of much debate. Combination therapy involves using more than one drug with different mechanisms of action. Patients at risk of or in visceral metastasis typically receive combination regimens [40]. Ghebeh and colleagues postulated that durvalumab and paclitaxel are safe and efficacious when administered in phase I/II studies conducted at a single institution, single-arm, and open-label for metastatic TNBC [126]. Adding durvalumab to paclitaxel is safe, allowing additional agents to be added.

Furthermore, the combined delivery of siRNA and a chemotherapeutic agent via nanotechnology can offer several advantages, including gene silencing and evading the P-glycoprotein efflux pump [127]. These advantages in combination therapies are evident in a study conducted by Wan et al. where a A2780/CisR-resistant xenograft tumor and LCC-6MDR multidrug-resistant breast cancer orthotopic model demonstrated superior antitumor activity with co-loaded PTX/Cisplatin drug micelles compared with single drug micelles [128]. As a result of co-loading in the micelles, both drugs are released slowly into the serum, their pharmacokinetics improve, and their tumor distribution is increased.

Combination therapy in breast cancer therapy does not always translate into increased chances of more toxicities, as would be assumed. Berko et al. demonstrated that combinatorial treatment might benefit cancer treatment while reducing toxicity risk [129]. In this study, dispersion polymerization was used to design stealth polymeric nanoparticles loaded with paclitaxel and 17-AAG. Two breast tumor cell lines (MCF-7 and SKBR-3) were tested for nanoparticle cytotoxicity. Paclitaxel (free drug), paclitaxel-17AAG combination (free drug), and dual drug-loaded nanoparticles had similar cytotoxic outcomes on both cell lines. The combination of paclitaxel and 17-AAG resulted in a synergistic effect, with paclitaxel’s concentration being half that in combination with 17-AAG and cytotoxicity being the same. The dose of paclitaxel was decreased without compromising its therapeutic effectiveness. Moreover, a prospective, single-center, phase II trial conducted by Ban et al. administered docetaxel and oxaliplatin every three weeks for six cycles to chemotherapy-naive non-small cell lung cancer patients. Results reveal that oxaliplatin and docetaxel combined are effective for patients with minimal side effects [130].

## 5. Challenges in TNBC Treatment

Generally, TNBC is more aggressive and has poorer outcomes than other breast cancer subtypes [57,131]. Despite significant progress, TNBC treatment still faces some challenges, and current treatments possess limitations. Foremost, as opposed to hormone receptor-positive breast cancers, TNBC does not have specific target receptors, such as estrogen receptors, progesterone receptors, and HER2, that can be targeted with specific therapies. As a result, targeting therapies can be challenging, resulting in limited treatment options; hence, chemotherapy remains the mainstay of treatment [132,133]. Despite its effectiveness, chemotherapy has significant side effects, toxicity, and varying patient treatment outcomes [76]. Multifactorial resistance mechanisms include genetic alterations, microenvironmental changes, and cancer stem cells [60]. The key to improving treatment outcomes is overcoming chemotherapy resistance.

In addition, equated to other breast cancer subtypes, TNBC has higher recurrence and distant metastasis rates [49]. It is still possible for a recurrence even if the initial treatment is successful. To prevent the recurrence and metastatic spread of the disease, it is imperative to develop more effective treatments. Patients with advanced or metastatic disease have fewer treatment options. On top of that, TNBC has a high degree of heterogeneity, multiple subtypes with distinct molecular profiles [131]. However, targeted therapies are lacking for these subtypes [134]. Personalized treatment approaches are limited for these subtypes due to the limited number of effective therapies available. Identifying novel therapeutic targets, developing targeted therapies, and improving the understanding of tumor heterogeneity are all required to address these limitations. Developing treatment options for TNBC requires collaboration among researchers, clinicians, and pharmaceutical companies. Recent research on TNBC treatment advocates that nanotechnology offers several potential solutions to tackle these issues of sub-optimal TNBC therapy. Table 2 below shows the drawbacks associated with TNBC therapy. Table 2 provides a summary of limitations and toxicities encountered in the current TNBC treatments.

## 6. Future Perspective

### 6.1. Drugs in Clinical Trial

TNBC is a great burden to society, especially in countries with a high population of black and brown women. There are currently clinical trials and research activities on new drugs and targeted therapies based on molecular characterization to improve pCR and survival in TNBC [71]. Instead of only using systemic chemotherapy for mTNBC, personalized medicine could be used. It is important that each patient receives a treatment plan that is individualized according to the unique circumstances of their TNBC. New treatments for TNBC are being tested in clinical trials. It may be possible for patients who qualify for clinical trials to receive treatments that are not yet available to the public. Based on positive results from bench works, cisplatin and gemcitabine should be studied in clinical trials to determine their efficacy in treating breast cancers associated with BRCA1 and triple negative. A multicenter, randomized, open-label phase 3 study conducted by Wang and his team compared low-dose capecitabine maintenance with observation following standard adjuvant therapy for early-stage TNBC [141]. In this study, disease-free survival was the primary endpoint. As secondary endpoints, future locoregional recurrence-free survival, overall survival, and adverse events were examined. Based on the results, low-dose capecitabine maintenance therapy resulted in significantly better 5-year disease-free survival. Another phase III trial comparing single platinum-based chemotherapy to capecitabine is being conducted in the US with patients who have residual TNBC after standard neoadjuvant chemotherapy [14]. Invasive disease-free survival is the primary objective.

### 6.2. Natural Agents

The percentage of anti-tumor drugs derived from natural products is approximately 50%. They can be natural products or semi-synthetic products. Taxanes, for example, are plant-based antitumor drugs currently in the clinic. Chemoprevention and chemotherapy can be achieved with natural products since they inhibit cell proliferation, regulate the cell cycle, and affect several signal pathways that lead to tumor growth [142]. Natural medicinal compounds have been broadly studied in cancer models, including breast cancer, for their anti-neoplastic properties. Withaferin-A (WA), a phytochemical in an ayurvedic plant called *Withania somnifera*, is known for its anti-inflammatory and anticancer properties. The combination of WA with chemotherapy has been shown to enhance chemotherapy efficacy in multiple in vitro and in vivo experimental models of TNBC [55].

### 6.3. Nanotechnology-Based Outlook

A combination of chemotherapy, surgery, and radiation is the most common cancer treatment. The drawbacks of these methods include lack of specificity and toxicity. Modern medicine aims to optimize drug efficacy and minimize side effects. The application of nanotechnology to cancer treatment can overcome conventional methods’ constraints by increasing the drug’s local concentration at cancer sites while reducing its concentration elsewhere. Nanotechnology can also reduce the dose of the drug required for a therapeutic effect and boost concentrations on cancer sites without harming healthy cells. Nanoparticle-based drug delivery systems include nanodiscs, polymeric nanoparticles, liposomes, and gold nanoparticles. Some of these have received FDA approval. In cancer therapy, nano-drugs may have a lot of potential due to their unique properties, including minimizing damage to healthy cells, overcoming multidrug resistance, and improving drug solubility [143].

In addition to the drug resistance crisis, which is on the verge of becoming a public health crisis, many current medications for TNBC cause adverse effects. There is a direct correlation between these adverse effects and treatment outcomes [28]. A debilitating side effect of neurotoxic cancer treatments such as taxanes and platinum agents is chemotherapy-induced peripheral neuropathy [139]. Hence, FDA-approved nanomedicines such as nab-paclitaxel, a nanoparticle albumin-bound paclitaxel formulation, have been designed to avoid neuropathy and hypersensitivity reactions associated with free paclitaxel formulation [81]. Through enhanced permeability and retention (EPR), nanomedicine can reduce systemic toxicity and be more effective for cancer therapy than conventional medicine [144]. Benefits of nanotechnology include (1) improvement in the delivery of poorly water-soluble drugs, (2) targeted delivery of the therapeutic agent, (3) co-delivery of two or more therapeutic agents for combination therapy, (4) improvement of the half-life of the drug in the circulation, (5) controlled and sustained release of the drug, (6) reduced multi-drug resistance, and (7) multimodality treatment by co-delivery of chemotherapeutics, radiotherapeutics, chemotherapeutics, and biotherapeutics to achieve a synergistic effect [22,46,143].

Improved cellular uptake of drugs is one of the most promising applications of nanotechnology. A study comparing free oxaliplatin with oxaliplatin loaded in polycaprolactone (PCL) nanoparticles was conducted to identify a more effective approach for treating non-small cell lung cancer [145]. In this study, compared with free oxaliplatin, PCL nanoparticles showed significantly greater uptake and efficacy in SK-MES-1 cells after 4 h (*p* < 0.05). DNA nanostructures have been demonstrated to perform various biomedical functions, such as drug delivery, imaging, biosensing, and theragnostic. Doxorubicin’s lack of precise targeting is one of its limitations in cancer medicines. In a study, researchers created DNA nano vehicles that contained doxorubicin at their cores and cetuximab on their surfaces to specifically target EGFR and the Watson–Crick base pairing. According to the results, cetuximab-conjugated DNA nanostructures enhance breast cancer cell targeting, improving overall efficiency in killing cancer cells [146]. External methods such as magnetism, ultrasound, and heat should be considered to enhance tumor uptake of these nanocarriers. There is potential for improving ADC through nanotechnology. In the study by Fisusi et al., the nanotechnology potential of PEG conjugation with monoclonal antibodies is exploited to improve their functionality [118]. PEG-monoclonal antibody conjugates were created by attaching a suitable functionalized polymer to the protein molecule without interfering with its binding site.

#### 6.3.1. Nanotechnology-Based Drug Targeting

**Figure 4:**
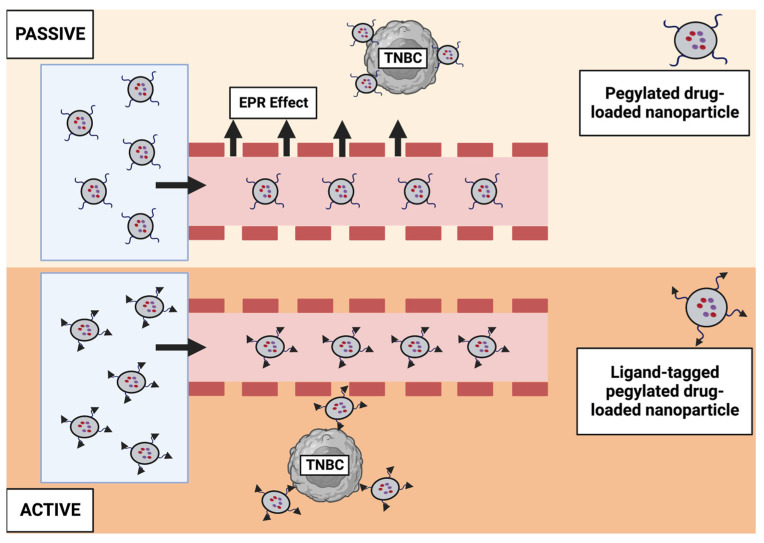
Targeting TNBC with passive and active strategies based on nanotechnology. Passive targeting relies on rapid vascularization growth and poor lymphatic drainage to increase permeability and retention (EPR). As a result, nanoparticles are retained in tumors. An active targeting strategy involves the interaction of nanoparticle surface ligands with cell surface receptors [147]. Created with Biorender.com, accessed on 16 June 2023.

Two major drug-targeting mechanisms exist: passive and active [147], (Figure 4). Passive targeting is based on EPR effect, caused by rapidly growing leaky vascularization and defective lymphatic drainage that cause nanoparticles and submicron particles to be retained in tumors. It has been extensively explored to show how nanoscale drug carriers can be used for cancer chemotherapy, including liposomes, dendrimers, polymeric micelles, polymer-drug conjugates, and inorganic nanoparticles. In addition to passing through hyper-permeable blood vessels, these nanoparticles accumulate at tumor sites through their EPR effect because of their small size [143].

##### Active Targeting

Even though passive targeting is crucial to transport drug molecules to the active sites, it is not enough, in this case, to ensure internalization into tumor tissues. Compared to passive targeting, this approach enhances cancer cell specificity and efficacy. In nanomedicine, active targeting strategies exploit specific biomolecular interactions between nanoparticle surface ligands and cell surface receptors or employ stimuli-sensitive drug carriers (colloids) engineered to experience modifications in their structure and physical properties under small changes in the environment, leading to triggered drug release specifically at the tumor site. Active targeting has two underlying rationales when compared to passive targeting: (1) increased retention of passively accumulated nanoparticles at diseased sites due to specific interactions between surface ligands and cell surface receptors; and (2) increased specific interactions between nanoparticles and diseased cells while minimizing non-targeted interactions [148].

This drug delivery strategy has emerged as a valuable approach to reaching the specific site of interest while avoiding systemic adverse effects. The specific recognition of the biological target through molecular recognition processes (ligand–receptor or antibody–antigen interactions) can be made possible by chemical conjugation of the drug delivery system’s surface to target tissue ligands. Typically, this approach leads to receptor-mediated cell internalization. The treatment of tumors has involved the use of several agents. These include monoclonal antibodies, peptides, aptamers, integrins, folate groups, and transferrins. Another type of active targeting involves stimuli-sensitive carriers. These carriers experience rapid changes in their structure and physical properties (disruption/aggregation, swelling/deswelling, etc.) under exposure to small environmental modifications. These changes are usually reversible and allow the polymer/carrier to return to its initial state upon removal of the stimulus. Current environmentally responsive drug delivery systems include a physical response (temperature, light, magnetic, ultrasonic, etc.), a chemical response (pH, reduction), and a biological response (enzyme, glucose) [149].

In active targeting, ligands, such as antibodies or peptides, bind specifically to cancer cell receptors [150]. Concerning targeting TNBC, various discoveries of antibodies and peptide–drug conjugates have been investigated, and the findings indicate that peptide–drug conjugates can be harnessed for therapeutics in TNBC management [151,152,153]. For instance, Demeule et al. highlighted sortilin (SORT1), also known as neurotensin receptor-3, a membrane-bound receptor within the vacuolar protein sorting 10 proteins (VPS10P) family, which is expressed in 59% of TNBC [152]. Since there are no specific targets for patients with TNBC, multiple strategies have been explored to specifically target and exploit the SORT1 receptor. Using a peptide (TH19P01) conjugated to docetaxel, Demeule and his team found that the peptide–drug conjugate was internalized via the SORT1 receptors and exerted in vivo and in vitro antiproliferative effects on SORT1-positive TNBC. Another study showed that the F7AK3 antibody binds simultaneously to trophoblast cell surface antigen 2 (TROP2) and CD3 antigens in TNBC cell lines, causing T cell activation and cytotoxicity [153]. As part of this study, F7AK3 was evaluated in vitro and in vivo against TNBC cells in a xenograft model. The results indicate that F7AK3 has potent antitumor activity against TNBC cells, suggesting it should be further studied and clinically evaluated for advanced TNBC patients.

##### Passive Targeting

Various approaches have been studied to target drugs using nanoparticles. Passive targeting is as fundamental to drug delivery as active targeting. Passive targeting is a targeting approach where a drug or therapeutic agent is designed to accumulate selectively in a target tissue or organ without active intervention [154]. This approach is used in various fields, such as drug delivery, imaging, and cancer therapy.

EPR effect: This is a common method of passively targeting cancer cells. It has been revealed that the tumor vasculature is leaky, allowing macromolecules and nanoparticles to accumulate. The accumulation is due to the EPR effect, where leaky blood vessels allow 10–200 nm particles to enter the tumor interstitial [155]. Following the intravenous delivery of nanoscale materials, Matsumura and Maeda published in 1986 two key findings that formed the basis of passive nanoparticle targeting [156]. A wide range of preclinical studies has supported these findings. First, macromolecular drug carriers spontaneously accumulated in solid tumors with leaky vasculature. Secondly, nanoparticles are retained intratumorally due to a compromised lymphatic drainage system. Nanoparticles penetrate tumors, but not healthy tissue layers, due to the abnormal vasculatures in neoplastic tissues. By incorporating a passive drug delivery approach, it is possible to improve the effectiveness of chemotherapeutic agents with negligible adverse effects by loading them inside nano-carrier structures [157]. The EPR effect is due to a solid tumor’s unique anatomical and pathophysiological characteristics. Most solid tumors have a higher vascular density than normal tissues; that is angiogenesis, a critical feature of tumors to sustain their rapid growth. Most solid tumors possess defective architecture, such as large gaps between endothelial cells and no smooth muscle layers [158]. Hence, macromolecules easily extravasate the tumor blood vessels and accumulate selectively in neoplastic tissues [159]. The effect of EPR extends beyond passive targeting. It also means prolonged drug retention for weeks.

Aside from factors related to tumor microvasculature, nanoparticle delivery is also affected by its size, shape, and surface charge. The size of developed nanomedicines is paramount because it differentiates between nanoparticles that can rapidly leak into capillaries and those that can be cleared by the macrophages of the reticuloendothelial system [160]. ‘Proper’-sized nanoparticles can easily pass through the gaps in endothelial linings of leaky solid tumors. Regarding surface characteristics, any drug carrier should ideally have a hydrophilic surface to retard macrophage capture. This can be actualized by (i) coating their surface with a hydrophilic polymer, such as polyethylene glycol (PEG) or Poly(N-(2-hydroxypropyl) methacrylamide) (PHPMA); this stealth layer protects the nanoparticles from opsonization by repelling plasma proteins; and, alternatively, (ii) the drug delivery system can be made of block copolymers with hydrophilic and hydrophobic domains. While passive targeting techniques have shown promising results in improving cancer drug delivery, alternative approaches and techniques are available to enhance drug delivery to neoplastic tissues.

## 7. Concluding Remarks

Recently, TNBC has received a lot of attention. There have been numerous attempts to improve treatment for this form of breast cancer, which stands as the most aggressive. TNBC is currently treated with chemotherapy, surgery, and radiotherapy, with chemotherapy being the most common method. The aim of chemotherapy is to deliver cytotoxic agents to the tumor site while sparing healthy cells from toxicity. However, there have been many challenges to achieving this goal, including chemoresistance and off-target toxicity. The field of cancer nanotechnology has attracted a lot of attention, which has led to new drug discoveries and delivery systems for the treatment of cancer. The benefits of delivering chemotherapeutics can be realized through active and passive targeting. Despite its outstanding advantages for cancer management, nanotechnology has its drawbacks, primarily resource intensiveness and unproven reliability and applicability. These limitations must be continuously improved for the platform to be used in clinical decision-making.

## Figures and Tables

**Table 1:** Prognostic biomarkers in TNBC.

No.	Prognostic Biomarkers	Ref.
1	Estrogen receptor (ER) and progesterone receptor (PR) status	[46]
2	Cathepsin D	[7]
3	Mutations of p53	[44]
4	Epidermal growth factor receptor (EGFR)	[11]
5	BRCA 1/2 germline mutation	[37]
6	Vascular endothelial growth factor (VEGF)	[47]
7	Notch signaling pathway	[48]
8	Tumor-infiltrating lymphocytes (TILs)	[28]
9	Androgen receptor	[49]
10	Ki67 index	[50]

**Table 2:** Limitations and toxicities associated with current treatment for TNBC.

Class	Examples	Limitation/Toxicities	Ref.
**Surgery**	Lumpectomy Mastectomy	Residual disease that recurs or metastasizes Limited effectiveness in advanced stages Surgical complications/impact on cosmesis	[135]
**Radiation**	External beam radiation therapy IORT	Myelosuppression Skin reactions Radiation pneumonitis Lymphedema	[136]
**Anthracyclines**	Doxorubicin Epirubicin	Cardiotoxicity Bone marrow suppression Cumulative dose limitations Drug resistance Limited efficacy in metastatic disease Risk of secondary malignancies	[40,75,137]
**Taxanes**	Paclitaxel Docetaxel Cabazitaxel	Drug resistance Bone marrow suppression Peripheral neuropathy Gastrointestinal issues Hypersensitivity reactions	[53,76,80]
**Platinum**	Cisplatin Carboplatin Oxaliplatin	Bone marrow suppression Kidney damage Peripheral neuropathy Allergic reactions Gastrointestinal issues	[138,139]
**PARP Inhibitors**	Olaparib Talazoparib	Immunosuppression-induced sepsis Myelodysplastic syndrome Acute myeloid leukemia Fatigue Anemia Drug resistance	[97,140]
**P13/AKT** **/mTOR Inhibitors**	Ipatasertib Everolimus	Pulmonary toxicity Elevated liver enzyme Mucositis Hyperglycemia	[123]

## Data Availability

Not applicable.
